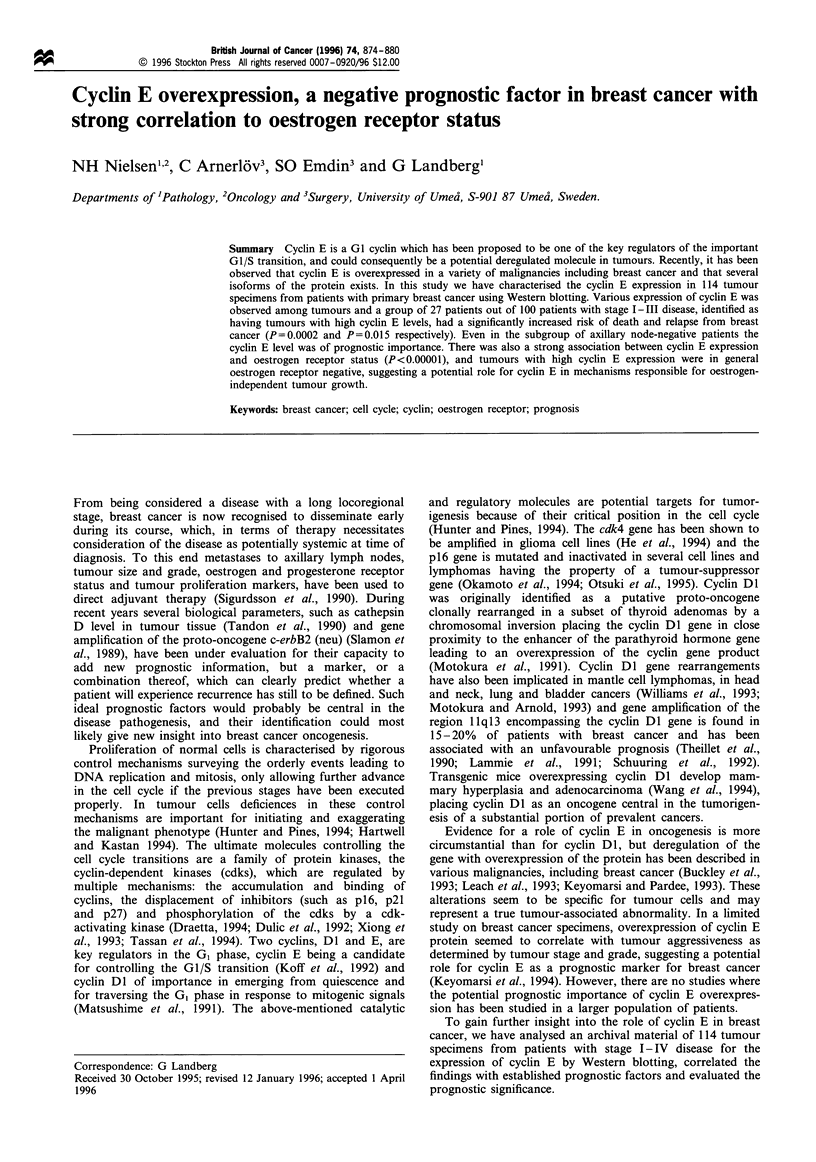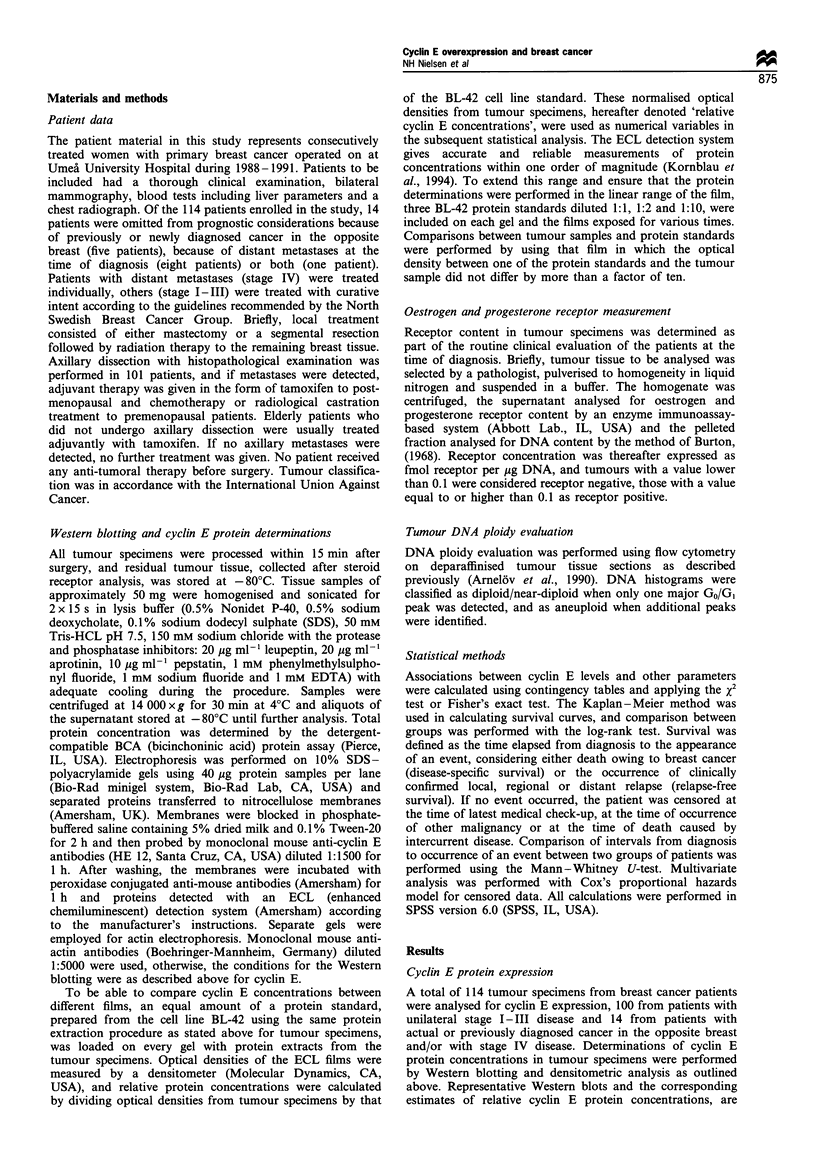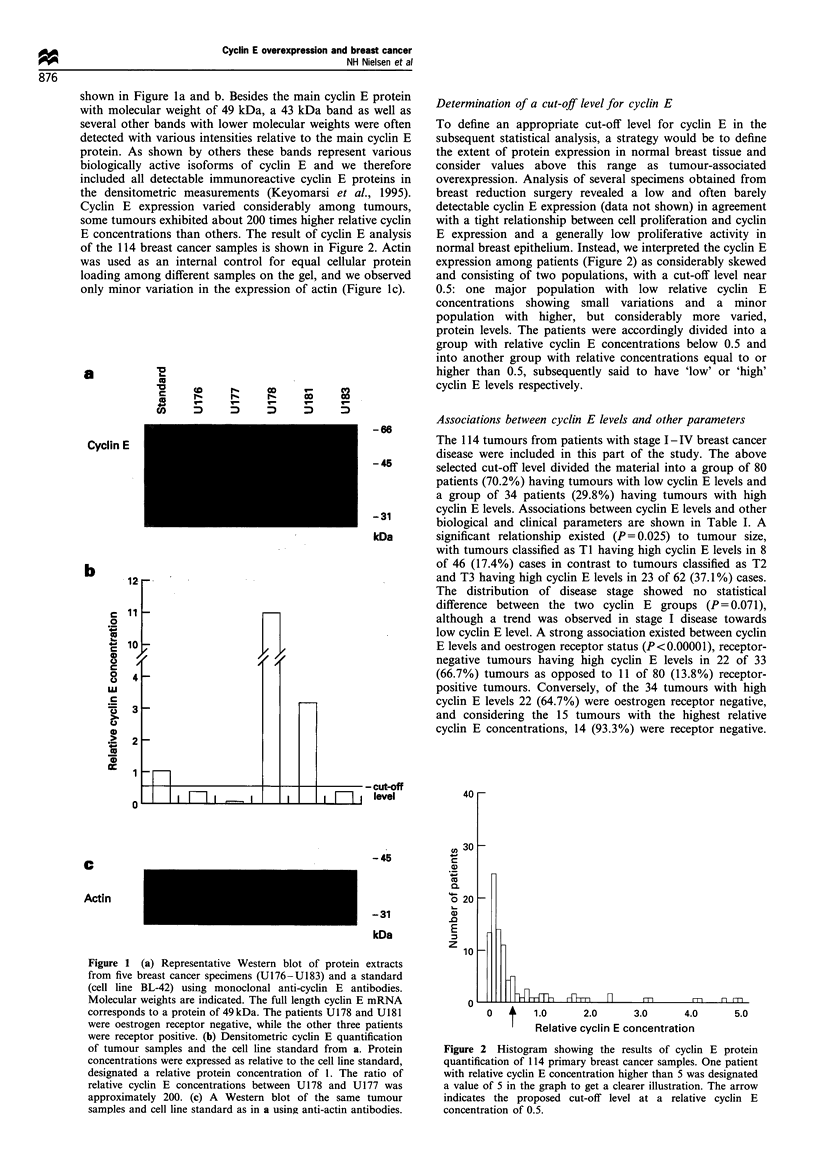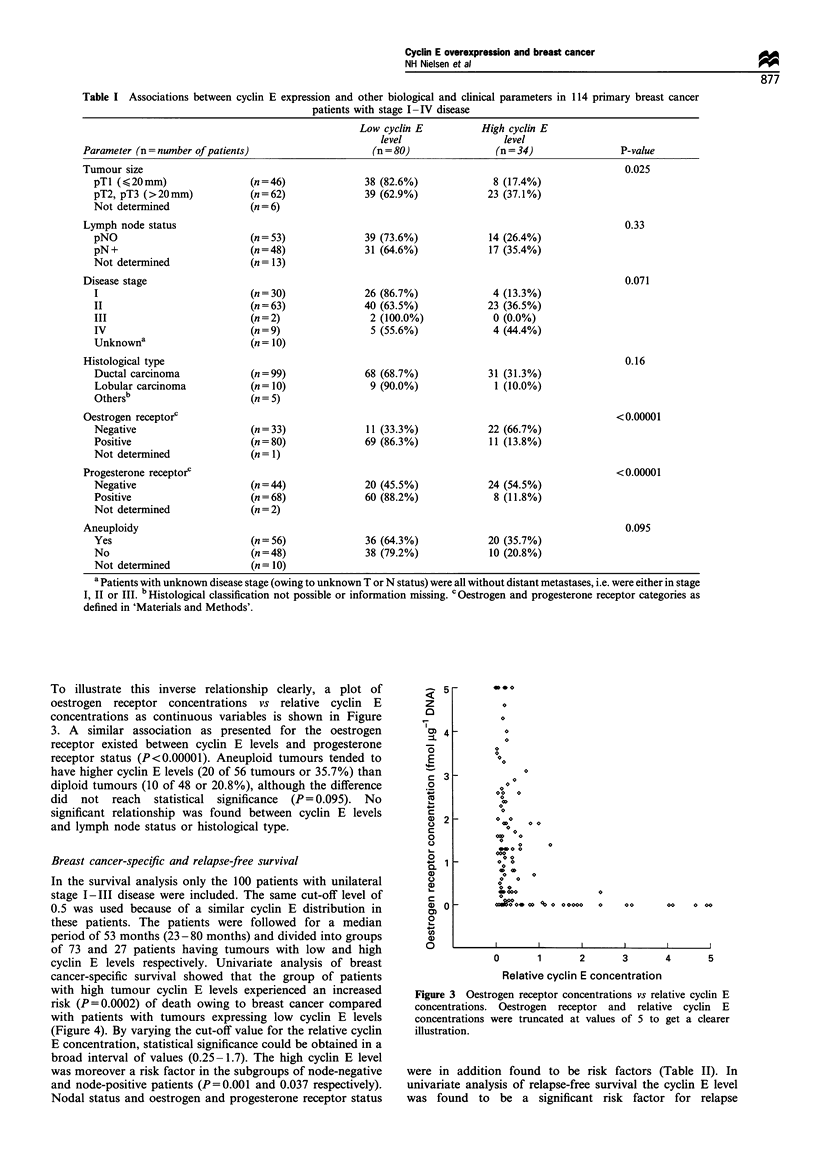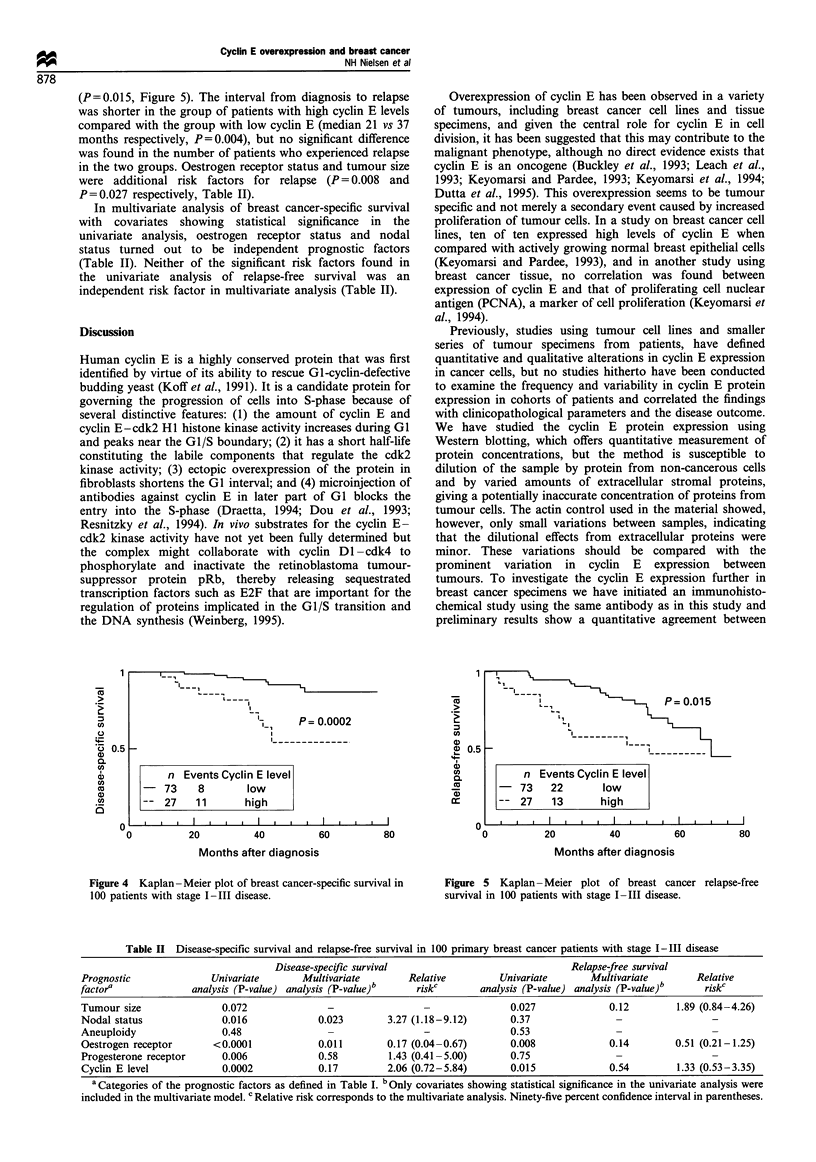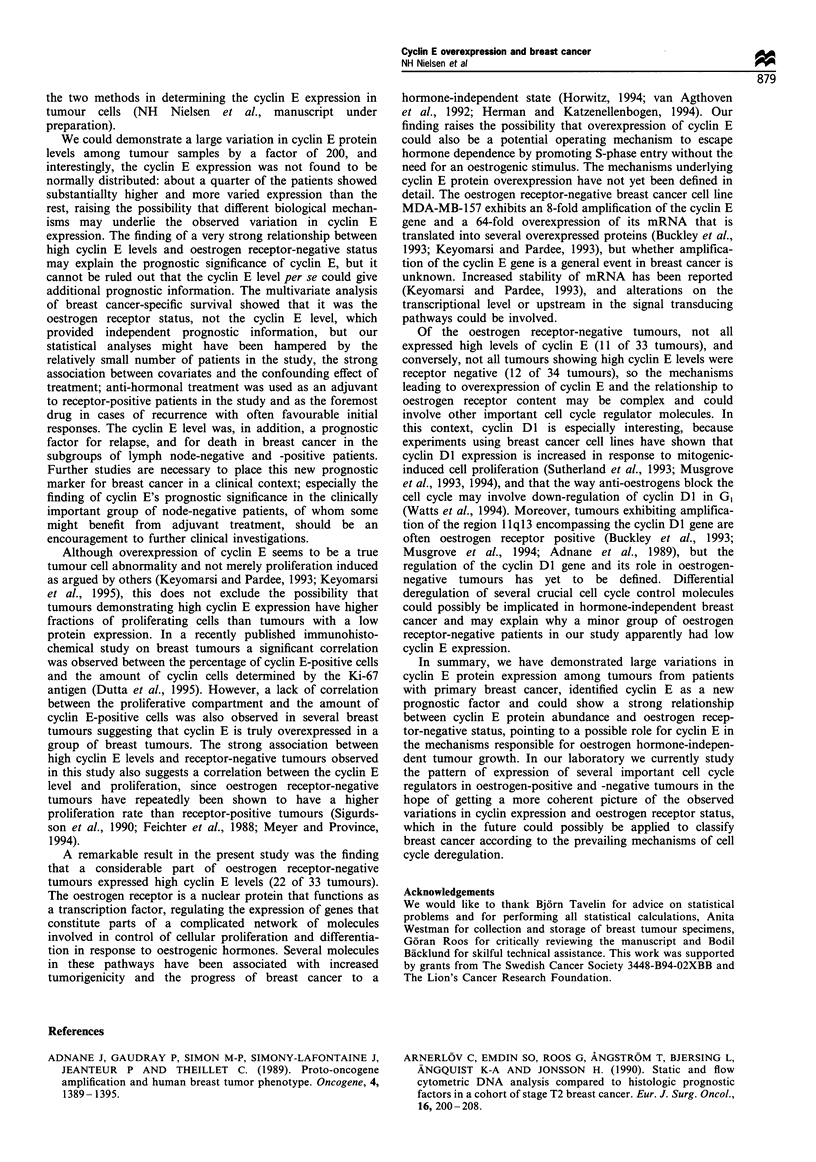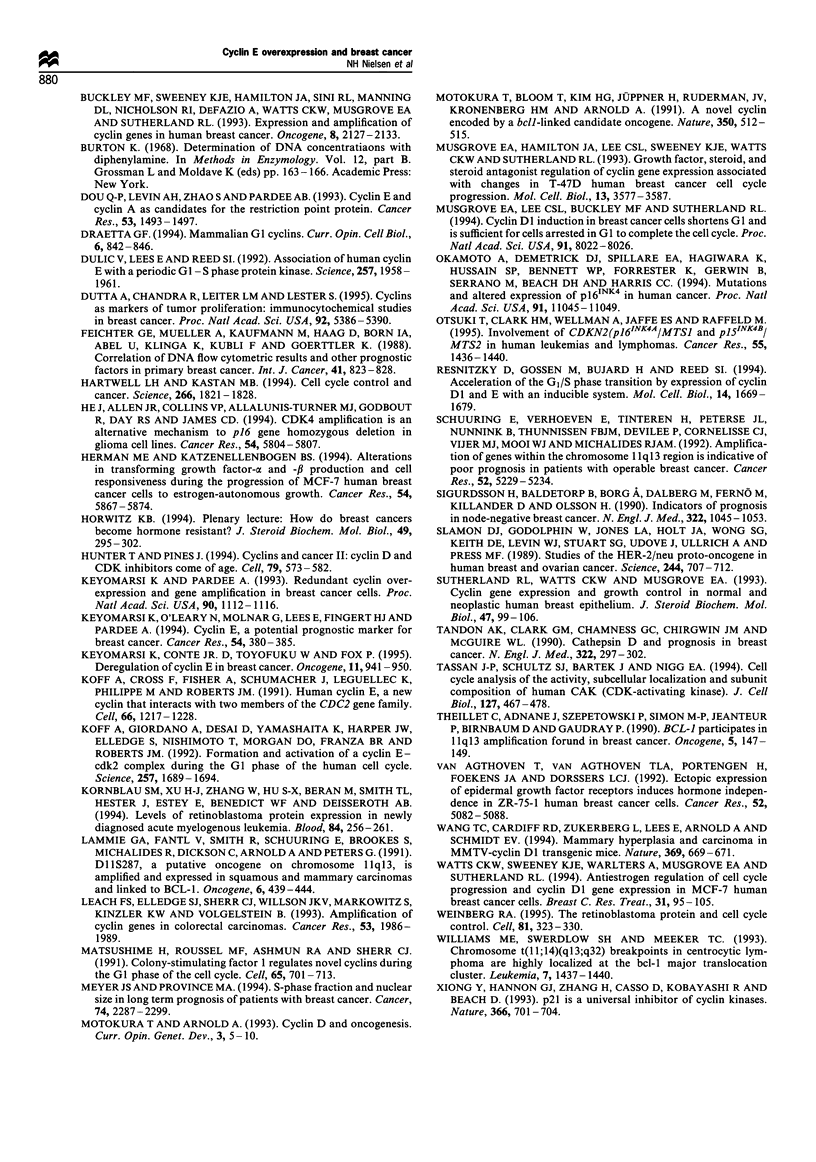# Cyclin E overexpression, a negative prognostic factor in breast cancer with strong correlation to oestrogen receptor status.

**DOI:** 10.1038/bjc.1996.451

**Published:** 1996-09

**Authors:** N. H. Nielsen, C. Arnerlöv, S. O. Emdin, G. Landberg

**Affiliations:** Department of Pathology, University of Umeå, Sweden.

## Abstract

**Images:**


British Journal of Cancer (1996) 74, 874-880
© 1996 Stockton Press All rights reserved 0007-0920/96 $12.00

### Cyclin E overexpression, a negative prognostic factor in breast cancer with strong correlation to oestrogen receptor status

NH Nielsen[1,2], C Arnerlöv[3], SO Emdin[3] and G Landberg[1]

*Departments of [1]Pathology, [2]Oncology and [3]Surgery, University of Umeå, S-901 87 Umeå, Sweden.*

**Summary** Cyclin E is a G1 cyclin which has been proposed to be one of the key regulators of the important G1/S transition, and could consequently be a potential deregulated molecule in tumours. Recently, it has been observed that cyclin E is overexpressed in a variety of malignancies including breast cancer and that several isoforms of the protein exists. In this study we have characterised the cyclin E expression in 114 tumour specimens from patients with primary breast cancer using Western blotting. Various expression of cyclin E was observed among tumours and a group of 27 patients out of 100 patients with stage I-III disease, identified as having tumours with high cyclin E levels, had a significantly increased risk of death and relapse from breast cancer ($P=0.0002$ and $P=0.015$ respectively). Even in the subgroup of axillary node-negative patients the cyclin E level was of prognostic importance. There was also a strong association between cyclin E expression and oestrogen receptor status ($P<0.00001$), and tumours with high cyclin E expression were in general oestrogen receptor negative, suggesting a potential role for cyclin E in mechanisms responsible for oestrogen-independent tumour growth.

**Keywords:** breast cancer; cell cycle; cyclin; oestrogen receptor; prognosis

From being considered a disease with a long locoregional stage, breast cancer is now recognised to disseminate early during its course, which, in terms of therapy necessitates consideration of the disease as potentially systemic at time of diagnosis. To this end metastases to axillary lymph nodes, tumour size and grade, oestrogen and progesterone receptor status and tumour proliferation markers, have been used to direct adjuvant therapy (Sigurdsson *et al.*, 1990). During recent years several biological parameters, such as cathepsin D level in tumour tissue (Tandon *et al.*, 1990) and gene amplification of the proto-oncogene c-*erb*B2 (neu) (Slamon *et al.*, 1989), have been under evaluation for their capacity to add new prognostic information, but a marker, or a combination thereof, which can clearly predict whether a patient will experience recurrence has still to be defined. Such ideal prognostic factors would probably be central in the disease pathogenesis, and their identification could most likely give new insight into breast cancer oncogenesis.

Proliferation of normal cells is characterised by rigorous control mechanisms surveying the orderly events leading to DNA replication and mitosis, only allowing further advance in the cell cycle if the previous stages have been executed properly. In tumour cells deficiences in these control mechanisms are important for initiating and exaggerating the malignant phenotype (Hunter and Pines, 1994; Hartwell and Kastan 1994). The ultimate molecules controlling the cell cycle transitions are a family of protein kinases, the cyclin-dependent kinases (cdks), which are regulated by multiple mechanisms: the accumulation and binding of cyclins, the displacement of inhibitors (such as p16, p21 and p27) and phosphorylation of the cdks by a cdk-activating kinase (Draetta, 1994; Dulic *et al.*, 1992; Xiong *et al.*, 1993; Tassan *et al.*, 1994). Two cyclins, D1 and E, are key regulators in the $G_1$ phase, cyclin E being a candidate for controlling the G1/S transition (Koff *et al.*, 1992) and cyclin D1 of importance in emerging from quiescence and for traversing the $G_1$ phase in response to mitogenic signals (Matsushime *et al.*, 1991). The above-mentioned catalytic and regulatory molecules are potential targets for tumorigenesis because of their critical position in the cell cycle (Hunter and Pines, 1994). The *cdk*4 gene has been shown to be amplified in glioma cell lines (He *et al.*, 1994) and the p16 gene is mutated and inactivated in several cell lines and lymphomas having the property of a tumour-suppressor gene (Okamoto *et al.*, 1994; Otsuki *et al.*, 1995). Cyclin D1 was originally identified as a putative proto-oncogene clonally rearranged in a subset of thyroid adenomas by a chromosomal inversion placing the cyclin D1 gene in close proximity to the enhancer of the parathyroid hormone gene leading to an overexpression of the cyclin gene product (Motokura *et al.*, 1991). Cyclin D1 gene rearrangements have also been implicated in mantle cell lymphomas, in head and neck, lung and bladder cancers (Williams *et al.*, 1993; Motokura and Arnold, 1993) and gene amplification of the region 11q13 encompassing the cyclin D1 gene is found in 15-20% of patients with breast cancer and has been associated with an unfavourable prognosis (Theillet *et al.*, 1990; Lammie *et al.*, 1991; Schuuring *et al.*, 1992). Transgenic mice overexpressing cyclin D1 develop mammary hyperplasia and adenocarcinoma (Wang *et al.*, 1994), placing cyclin D1 as an oncogene central in the tumorigenesis of a substantial portion of prevalent cancers.

Evidence for a role of cyclin E in oncogenesis is more circumstantial than for cyclin D1, but deregulation of the gene with overexpression of the protein has been described in various malignancies, including breast cancer (Buckley *et al.*, 1993; Leach *et al.*, 1993; Keyomarsi and Pardee, 1993). These alterations seem to be specific for tumour cells and may represent a true tumour-associated abnormality. In a limited study on breast cancer specimens, overexpression of cyclin E protein seemed to correlate with tumour aggressiveness as determined by tumour stage and grade, suggesting a potential role for cyclin E as a prognostic marker for breast cancer (Keyomarsi *et al.*, 1994). However, there are no studies where the potential prognostic importance of cyclin E overexpression has been studied in a larger population of patients.

To gain further insight into the role of cyclin E in breast cancer, we have analysed an archival material of 114 tumour specimens from patients with stage I-IV disease for the expression of cyclin E by Western blotting, correlated the findings with established prognostic factors and evaluated the prognostic significance.

Correspondence: G Landberg
Received 30 October 1995; revised 12 January 1996; accepted 1 April 1996

### Materials and methods

### *Patient data*

The patient material in this study represents consecutively treated women with primary breast cancer operated on at Umeå University Hospital during 1988–1991. Patients to be included had a thorough clinical examination, bilateral mammography, blood tests including liver parameters and a chest radiograph. Of the 114 patients enrolled in the study, 14 patients were omitted from prognostic considerations because of previously or newly diagnosed cancer in the opposite breast (five patients), because of distant metastases at the time of diagnosis (eight patients) or both (one patient). Patients with distant metastases (stage IV) were treated individually, others (stage I–III) were treated with curative intent according to the guidelines recommended by the North Swedish Breast Cancer Group. Briefly, local treatment consisted of either mastectomy or a segmental resection followed by radiation therapy to the remaining breast tissue. Axillary dissection with histopathological examination was performed in 101 patients, and if metastases were detected, adjuvant therapy was given in the form of tamoxifen to post-menopausal and chemotherapy or radiological castration treatment to premenopausal patients. Elderly patients who did not undergo axillary dissection were usually treated adjuvantly with tamoxifen. If no axillary metastases were detected, no further treatment was given. No patient received any anti-tumoral therapy before surgery. Tumour classification was in accordance with the International Union Against Cancer.

### *Western blotting and cyclin E protein determinations*

All tumour specimens were processed within 15 min after surgery, and residual tumour tissue, collected after steroid receptor analysis, was stored at −80°C. Tissue samples of approximately 50 mg were homogenised and sonicated for 2×15 s in lysis buffer (0.5% Nonidet P-40, 0.5% sodium deoxycholate, 0.1% sodium dodecyl sulphate (SDS), 50 mM Tris-HCL pH 7.5, 150 mM sodium chloride with the protease and phosphatase inhibitors: 20 $\mu g\ ml^{-1}$ leupeptin, 20 $\mu g\ ml^{-1}$ aprotinin, 10 $\mu g\ ml^{-1}$ pepstatin, 1 mM phenylmethylsulphonyl fluoride, 1 mM sodium fluoride and 1 mM EDTA) with adequate cooling during the procedure. Samples were centrifuged at 14 000×*g* for 30 min at 4°C and aliquots of the supernatant stored at −80°C until further analysis. Total protein concentration was determined by the detergent-compatible BCA (bicinchoninic acid) protein assay (Pierce, IL, USA). Electrophoresis was performed on 10% SDS–polyacrylamide gels using 40 $\mu g$ protein samples per lane (Bio-Rad minigel system, Bio-Rad Lab, CA, USA) and separated proteins transferred to nitrocellulose membranes (Amersham, UK). Membranes were blocked in phosphate-buffered saline containing 5% dried milk and 0.1% Tween-20 for 2 h and then probed by monoclonal mouse anti-cyclin E antibodies (HE 12, Santa Cruz, CA, USA) diluted 1:1500 for 1 h. After washing, the membranes were incubated with peroxidase conjugated anti-mouse antibodies (Amersham) for 1 h and proteins detected with an ECL (enhanced chemiluminescent) detection system (Amersham) according to the manufacturer's instructions. Separate gels were employed for actin electrophoresis. Monoclonal mouse anti-actin antibodies (Boehringer-Mannheim, Germany) diluted 1:5000 were used, otherwise, the conditions for the Western blotting were as described above for cyclin E.

To be able to compare cyclin E concentrations between different films, an equal amount of a protein standard, prepared from the cell line BL-42 using the same protein extraction procedure as stated above for tumour specimens, was loaded on every gel with protein extracts from the tumour specimens. Optical densities of the ECL films were measured by a densitometer (Molecular Dynamics, CA, USA), and relative protein concentrations were calculated by dividing optical densities from tumour specimens by that of the BL-42 cell line standard. These normalised optical densities from tumour specimens, hereafter denoted 'relative cyclin E concentrations', were used as numerical variables in the subsequent statistical analysis. The ECL detection system gives accurate and reliable measurements of protein concentrations within one order of magnitude (Kornblau *et al*., 1994). To extend this range and ensure that the protein determinations were performed in the linear range of the film, three BL-42 protein standards diluted 1:1, 1:2 and 1:10, were included on each gel and the films exposed for various times. Comparisons between tumour samples and protein standards were performed by using that film in which the optical density between one of the protein standards and the tumour sample did not differ by more than a factor of ten.

### *Oestrogen and progesterone receptor measurement*

Receptor content in tumour specimens was determined as part of the routine clinical evaluation of the patients at the time of diagnosis. Briefly, tumour tissue to be analysed was selected by a pathologist, pulverised to homogeneity in liquid nitrogen and suspended in a buffer. The homogenate was centrifuged, the supernatant analysed for oestrogen and progesterone receptor content by an enzyme immunoassay-based system (Abbott Lab., IL, USA) and the pelleted fraction analysed for DNA content by the method of Burton, (1968). Receptor concentration was thereafter expressed as fmol receptor per $\mu g$ DNA, and tumours with a value lower than 0.1 were considered receptor negative, those with a value equal to or higher than 0.1 as receptor positive.

### *Tumour DNA ploidy evaluation*

DNA ploidy evaluation was performed using flow cytometry on deparaffinised tumour tissue sections as described previously (Arnelöv *et al*., 1990). DNA histograms were classified as diploid/near-diploid when only one major $G_0/G_1$ peak was detected, and as aneuploid when additional peaks were identified.

### *Statistical methods*

Associations between cyclin E levels and other parameters were calculated using contingency tables and applying the $\chi^2$ test or Fisher's exact test. The Kaplan–Meier method was used in calculating survival curves, and comparison between groups was performed with the log-rank test. Survival was defined as the time elapsed from diagnosis to the appearance of an event, considering either death owing to breast cancer (disease-specific survival) or the occurrence of clinically confirmed local, regional or distant relapse (relapse-free survival). If no event occurred, the patient was censored at the time of latest medical check-up, at the time of occurrence of other malignancy or at the time of death caused by intercurrent disease. Comparison of intervals from diagnosis to occurrence of an event between two groups of patients was performed using the Mann–Whitney *U*-test. Multivariate analysis was performed with Cox's proportional hazards model for censored data. All calculations were performed in SPSS version 6.0 (SPSS, IL, USA).

### Results

### *Cyclin E protein expression*

A total of 114 tumour specimens from breast cancer patients were analysed for cyclin E expression, 100 from patients with unilateral stage I–III disease and 14 from patients with actual or previously diagnosed cancer in the opposite breast and/or with stage IV disease. Determinations of cyclin E protein concentrations in tumour specimens were performed by Western blotting and densitometric analysis as outlined above. Representative Western blots and the corresponding estimates of relative cyclin E protein concentrations, are shown in Figure 1a and b. Besides the main cyclin E protein with molecular weight of 49 kDa, a 43 kDa band as well as several other bands with lower molecular weights were often detected with various intensities relative to the main cyclin E protein. As shown by others these bands represent various biologically active isoforms of cyclin E and we therefore included all detectable immunoreactive cyclin E proteins in the densitometric measurements (Keyomarsi *et al*., 1995). Cyclin E expression varied considerably among tumours, some tumours exhibited about 200 times higher relative cyclin E concentrations than others. The result of cyclin E analysis of the 114 breast cancer samples is shown in Figure 2. Actin was used as an internal control for equal cellular protein loading among different samples on the gel, and we observed only minor variation in the expression of actin (Figure 1c).

**Figure 1** (**a**) Representative Western blot of protein extracts from five breast cancer specimens (U176–U183) and a standard (cell line BL-42) using monoclonal anti-cyclin E antibodies. Molecular weights are indicated. The full length cyclin E mRNA corresponds to a protein of 49 kDa. The patients U178 and U181 were oestrogen receptor negative, while the other three patients were receptor positive. (**b**) Densitometric cyclin E quantification of tumour samples and the cell line standard from **a**. Protein concentrations were expressed as relative to the cell line standard, designated a relative protein concentration of 1. The ratio of relative cyclin E concentrations between U178 and U177 was approximately 200. (**c**) A Western blot of the same tumour samples and cell line standard as in **a** using anti-actin antibodies.

*Determination of a cut-off level for cyclin E*

To define an appropriate cut-off level for cyclin E in the subsequent statistical analysis, a strategy would be to define the extent of protein expression in normal breast tissue and consider values above this range as tumour-associated overexpression. Analysis of several specimens obtained from breast reduction surgery revealed a low and often barely detectable cyclin E expression (data not shown) in agreement with a tight relationship between cell proliferation and cyclin E expression and a generally low proliferative activity in normal breast epithelium. Instead, we interpreted the cyclin E expression among patients (Figure 2) as considerably skewed and consisting of two populations, with a cut-off level near 0.5: one major population with low relative cyclin E concentrations showing small variations and a minor population with higher, but considerably more varied, protein levels. The patients were accordingly divided into a group with relative cyclin E concentrations below 0.5 and into another group with relative concentrations equal to or higher than 0.5, subsequently said to have 'low' or 'high' cyclin E levels respectively.

*Associations between cyclin E levels and other parameters*

The 114 tumours from patients with stage I–IV breast cancer disease were included in this part of the study. The above selected cut-off level divided the material into a group of 80 patients (70.2%) having tumours with low cyclin E levels and a group of 34 patients (29.8%) having tumours with high cyclin E levels. Associations between cyclin E levels and other biological and clinical parameters are shown in Table I. A significant relationship existed ($P=0.025$) to tumour size, with tumours classified as T1 having high cyclin E levels in 8 of 46 (17.4%) cases in contrast to tumours classified as T2 and T3 having high cyclin E levels in 23 of 62 (37.1%) cases. The distribution of disease stage showed no statistical difference between the two cyclin E groups ($P=0.071$), although a trend was observed in stage I disease towards low cyclin E level. A strong association existed between cyclin E levels and oestrogen receptor status ($P<0.00001$), receptor-negative tumours having high cyclin E levels in 22 of 33 (66.7%) tumours as opposed to 11 of 80 (13.8%) receptor-positive tumours. Conversely, of the 34 tumours with high cyclin E levels 22 (64.7%) were oestrogen receptor negative, and considering the 15 tumours with the highest relative cyclin E concentrations, 14 (93.3%) were receptor negative.

**Figure 2** Histogram showing the results of cyclin E protein quantification of 114 primary breast cancer samples. One patient with relative cyclin E concentration higher than 5 was designated a value of 5 in the graph to get a clearer illustration. The arrow indicates the proposed cut-off level at a relative cyclin E concentration of 0.5.

**Table I** Associations between cyclin E expression and other biological and clinical parameters in 114 primary breast cancer patients with stage I–IV disease

| *Parameter* (n = *number of patients*) | | *Low cyclin E level* (n = *80*) | *High cyclin E level* (n = *34*) | P-*value* |
|---|---|---|---|---|
| Tumour size | | | | 0.025 |
| pT1 (≤20 mm) | (*n* = 46) | 38 (82.6%) | 8 (17.4%) | |
| pT2, pT3 (>20 mm) | (*n* = 62) | 39 (62.9%) | 23 (37.1%) | |
| Not determined | (*n* = 6) | | | |
| Lymph node status | | | | 0.33 |
| pNO | (*n* = 53) | 39 (73.6%) | 14 (26.4%) | |
| pN+ | (*n* = 48) | 31 (64.6%) | 17 (35.4%) | |
| Not determined | (*n* = 13) | | | |
| Disease stage | | | | 0.071 |
| I | (*n* = 30) | 26 (86.7%) | 4 (13.3%) | |
| II | (*n* = 63) | 40 (63.5%) | 23 (36.5%) | |
| III | (*n* = 2) | 2 (100.0%) | 0 (0.0%) | |
| IV | (*n* = 9) | 5 (55.6%) | 4 (44.4%) | |
| Unknown[a] | (*n* = 10) | | | |
| Histological type | | | | 0.16 |
| Ductal carcinoma | (*n* = 99) | 68 (68.7%) | 31 (31.3%) | |
| Lobular carcinoma | (*n* = 10) | 9 (90.0%) | 1 (10.0%) | |
| Others[b] | (*n* = 5) | | | |
| Oestrogen receptor[c] | | | | <0.00001 |
| Negative | (*n* = 33) | 11 (33.3%) | 22 (66.7%) | |
| Positive | (*n* = 80) | 69 (86.3%) | 11 (13.8%) | |
| Not determined | (*n* = 1) | | | |
| Progesterone receptor[c] | | | | <0.00001 |
| Negative | (*n* = 44) | 20 (45.5%) | 24 (54.5%) | |
| Positive | (*n* = 68) | 60 (88.2%) | 8 (11.8%) | |
| Not determined | (*n* = 2) | | | |
| Aneuploidy | | | | 0.095 |
| Yes | (*n* = 56) | 36 (64.3%) | 20 (35.7%) | |
| No | (*n* = 48) | 38 (79.2%) | 10 (20.8%) | |
| Not determined | (*n* = 10) | | | |

[a] Patients with unknown disease stage (owing to unknown T or N status) were all without distant metastases, i.e. were either in stage I, II or III. [b] Histological classification not possible or information missing. [c] Oestrogen and progesterone receptor categories as defined in 'Materials and Methods'.

To illustrate this inverse relationship clearly, a plot of oestrogen receptor concentrations *vs* relative cyclin E concentrations as continuous variables is shown in Figure 3. A similar association as presented for the oestrogen receptor existed between cyclin E levels and progesterone receptor status ($P < 0.00001$). Aneuploid tumours tended to have higher cyclin E levels (20 of 56 tumours or 35.7%) than diploid tumours (10 of 48 or 20.8%), although the difference did not reach statistical significance ($P = 0.095$). No significant relationship was found between cyclin E levels and lymph node status or histological type.

### *Breast cancer-specific and relapse-free survival*

In the survival analysis only the 100 patients with unilateral stage I–III disease were included. The same cut-off level of 0.5 was used because of a similar cyclin E distribution in these patients. The patients were followed for a median period of 53 months (23–80 months) and divided into groups of 73 and 27 patients having tumours with low and high cyclin E levels respectively. Univariate analysis of breast cancer-specific survival showed that the group of patients with high tumour cyclin E levels experienced an increased risk ($P = 0.0002$) of death owing to breast cancer compared with patients with tumours expressing low cyclin E levels (Figure 4). By varying the cut-off value for the relative cyclin E concentration, statistical significance could be obtained in a broad interval of values (0.25–1.7). The high cyclin E level was moreover a risk factor in the subgroups of node-negative and node-positive patients ($P = 0.001$ and 0.037 respectively). Nodal status and oestrogen and progesterone receptor status

**Figure 3** Oestrogen receptor concentrations *vs* relative cyclin E concentrations. Oestrogen receptor and relative cyclin E concentrations were truncated at values of 5 to get a clearer illustration.

were in addition found to be risk factors (Table II). In univariate analysis of relapse-free survival the cyclin E level was found to be a significant risk factor for relapse

($P = 0.015$, Figure 5). The interval from diagnosis to relapse was shorter in the group of patients with high cyclin E levels compared with the group with low cyclin E (median 21 *vs* 37 months respectively, $P = 0.004$), but no significant difference was found in the number of patients who experienced relapse in the two groups. Oestrogen receptor status and tumour size were additional risk factors for relapse ($P = 0.008$ and $P = 0.027$ respectively, Table II).

In multivariate analysis of breast cancer-specific survival with covariates showing statistical significance in the univariate analysis, oestrogen receptor status and nodal status turned out to be independent prognostic factors (Table II). Neither of the significant risk factors found in the univariate analysis of relapse-free survival was an independent risk factor in multivariate analysis (Table II).

### Discussion

Human cyclin E is a highly conserved protein that was first identified by virtue of its ability to rescue G1-cyclin-defective budding yeast (Koff *et al.*, 1991). It is a candidate protein for governing the progression of cells into S-phase because of several distinctive features: (1) the amount of cyclin E and cyclin E–cdk2 H1 histone kinase activity increases during G1 and peaks near the G1/S boundary; (2) it has a short half-life constituting the labile components that regulate the cdk2 kinase activity; (3) ectopic overexpression of the protein in fibroblasts shortens the G1 interval; and (4) microinjection of antibodies against cyclin E in later part of G1 blocks the entry into the S-phase (Draetta, 1994; Dou *et al.*, 1993; Resnitzky *et al.*, 1994). *In vivo* substrates for the cyclin E–cdk2 kinase activity have not yet been fully determined but the complex might collaborate with cyclin D1–cdk4 to phosphorylate and inactivate the retinoblastoma tumour-suppressor protein pRb, thereby releasing sequestrated transcription factors such as E2F that are important for the regulation of proteins implicated in the G1/S transition and the DNA synthesis (Weinberg, 1995).

Overexpression of cyclin E has been observed in a variety of tumours, including breast cancer cell lines and tissue specimens, and given the central role for cyclin E in cell division, it has been suggested that this may contribute to the malignant phenotype, although no direct evidence exists that cyclin E is an oncogene (Buckley *et al.*, 1993; Leach *et al.*, 1993; Keyomarsi and Pardee, 1993; Keyomarsi *et al.*, 1994; Dutta *et al.*, 1995). This overexpression seems to be tumour specific and not merely a secondary event caused by increased proliferation of tumour cells. In a study on breast cancer cell lines, ten of ten expressed high levels of cyclin E when compared with actively growing normal breast epithelial cells (Keyomarsi and Pardee, 1993), and in another study using breast cancer tissue, no correlation was found between expression of cyclin E and that of proliferating cell nuclear antigen (PCNA), a marker of cell proliferation (Keyomarsi *et al.*, 1994).

Previously, studies using tumour cell lines and smaller series of tumour specimens from patients, have defined quantitative and qualitative alterations in cyclin E expression in cancer cells, but no studies hitherto have been conducted to examine the frequency and variability in cyclin E protein expression in cohorts of patients and correlated the findings with clinicopathological parameters and the disease outcome. We have studied the cyclin E protein expression using Western blotting, which offers quantitative measurement of protein concentrations, but the method is susceptible to dilution of the sample by protein from non-cancerous cells and by varied amounts of extracellular stromal proteins, giving a potentially inaccurate concentration of proteins from tumour cells. The actin control used in the material showed, however, only small variations between samples, indicating that the dilutional effects from extracellular proteins were minor. These variations should be compared with the prominent variation in cyclin E expression between tumours. To investigate the cyclin E expression further in breast cancer specimens we have initiated an immunohistochemical study using the same antibody as in this study and preliminary results show a quantitative agreement between

**Figure 4** Kaplan–Meier plot of breast cancer-specific survival in 100 patients with stage I–III disease.

**Figure 5** Kaplan–Meier plot of breast cancer relapse-free survival in 100 patients with stage I–III disease.

**Table II** Disease-specific survival and relapse-free survival in 100 primary breast cancer patients with stage I–III disease

| | *Disease-specific survival* | | | *Relapse-free survival* | | |
|---|---|---|---|---|---|---|
| *Prognostic factor*[a] | *Univariate analysis (P-value)* | *Multivariate analysis (P-value)*[b] | *Relative risk*[c] | *Univariate analysis (P-value)* | *Multivariate analysis (P-value)*[b] | *Relative risk*[c] |
| Tumour size | 0.072 | – | – | 0.027 | 0.12 | 1.89 (0.84–4.26) |
| Nodal status | 0.016 | 0.023 | 3.27 (1.18–9.12) | 0.37 | – | – |
| Aneuploidy | 0.48 | – | – | 0.53 | – | – |
| Oestrogen receptor | <0.0001 | 0.011 | 0.17 (0.04–0.67) | 0.008 | 0.14 | 0.51 (0.21–1.25) |
| Progesterone receptor | 0.006 | 0.58 | 1.43 (0.41–5.00) | 0.75 | – | – |
| Cyclin E level | 0.0002 | 0.17 | 2.06 (0.72–5.84) | 0.015 | 0.54 | 1.33 (0.53–3.35) |

[a] Categories of the prognostic factors as defined in Table I. [b] Only covariates showing statistical significance in the univariate analysis were included in the multivariate model. [c] Relative risk corresponds to the multivariate analysis. Ninety-five percent confidence interval in parentheses.

the two methods in determining the cyclin E expression in tumour cells (NH Nielsen *et al.*, manuscript under preparation).

We could demonstrate a large variation in cyclin E protein levels among tumour samples by a factor of 200, and interestingly, the cyclin E expression was not found to be normally distributed: about a quarter of the patients showed substantiallty higher and more varied expression than the rest, raising the possibility that different biological mechanisms may underlie the observed variation in cyclin E expression. The finding of a very strong relationship between high cyclin E levels and oestrogen receptor-negative status may explain the prognostic significance of cyclin E, but it cannot be ruled out that the cyclin E level *per se* could give additional prognostic information. The multivariate analysis of breast cancer-specific survival showed that it was the oestrogen receptor status, not the cyclin E level, which provided independent prognostic information, but our statistical analyses might have been hampered by the relatively small number of patients in the study, the strong association between covariates and the confounding effect of treatment; anti-hormonal treatment was used as an adjuvant to receptor-positive patients in the study and as the foremost drug in cases of recurrence with often favourable initial responses. The cyclin E level was, in addition, a prognostic factor for relapse, and for death in breast cancer in the subgroups of lymph node-negative and -positive patients. Further studies are necessary to place this new prognostic marker for breast cancer in a clinical context; especially the finding of cyclin E's prognostic significance in the clinically important group of node-negative patients, of whom some might benefit from adjuvant treatment, should be an encouragement to further clinical investigations.

Although overexpression of cyclin E seems to be a true tumour cell abnormality and not merely proliferation induced as argued by others (Keyomarsi and Pardee, 1993; Keyomarsi *et al.*, 1995), this does not exclude the possibility that tumours demonstrating high cyclin E expression have higher fractions of proliferating cells than tumours with a low protein expression. In a recently published immunohistochemical study on breast tumours a significant correlation was observed between the percentage of cyclin E-positive cells and the amount of cyclin cells determined by the Ki-67 antigen (Dutta *et al.*, 1995). However, a lack of correlation between the proliferative compartment and the amount of cyclin E-positive cells was also observed in several breast tumours suggesting that cyclin E is truly overexpressed in a group of breast tumours. The strong association between high cyclin E levels and receptor-negative tumours observed in this study also suggests a correlation between the cyclin E level and proliferation, since oestrogen receptor-negative tumours have repeatedly been shown to have a higher proliferation rate than receptor-positive tumours (Sigurdsson *et al.*, 1990; Feichter *et al.*, 1988; Meyer and Province, 1994).

A remarkable result in the present study was the finding that a considerable part of oestrogen receptor-negative tumours expressed high cyclin E levels (22 of 33 tumours). The oestrogen receptor is a nuclear protein that functions as a transcription factor, regulating the expression of genes that constitute parts of a complicated network of molecules involved in control of cellular proliferation and differentiation in response to oestrogenic hormones. Several molecules in these pathways have been associated with increased tumorigenicity and the progress of breast cancer to a hormone-independent state (Horwitz, 1994; van Agthoven *et al.*, 1992; Herman and Katzenellenbogen, 1994). Our finding raises the possibility that overexpression of cyclin E could also be a potential operating mechanism to escape hormone dependence by promoting S-phase entry without the need for an oestrogenic stimulus. The mechanisms underlying cyclin E protein overexpression have not yet been defined in detail. The oestrogen receptor-negative breast cancer cell line MDA-MB-157 exhibits an 8-fold amplification of the cyclin E gene and a 64-fold overexpression of its mRNA that is translated into several overexpressed proteins (Buckley *et al.*, 1993; Keyomarsi and Pardee, 1993), but whether amplification of the cyclin E gene is a general event in breast cancer is unknown. Increased stability of mRNA has been reported (Keyomarsi and Pardee, 1993), and alterations on the transcriptional level or upstream in the signal transducing pathways could be involved.

Of the oestrogen receptor-negative tumours, not all expressed high levels of cyclin E (11 of 33 tumours), and conversely, not all tumours showing high cyclin E levels were receptor negative (12 of 34 tumours), so the mechanisms leading to overexpression of cyclin E and the relationship to oestrogen receptor content may be complex and could involve other important cell cycle regulator molecules. In this context, cyclin D1 is especially interesting, because experiments using breast cancer cell lines have shown that cyclin D1 expression is increased in response to mitogenic-induced cell proliferation (Sutherland *et al.*, 1993; Musgrove *et al.*, 1993, 1994), and that the way anti-oestrogens block the cell cycle may involve down-regulation of cyclin D1 in $G_1$ (Watts *et al.*, 1994). Moreover, tumours exhibiting amplification of the region 11q13 encompassing the cyclin D1 gene are often oestrogen receptor positive (Buckley *et al.*, 1993; Musgrove *et al.*, 1994; Adnane *et al.*, 1989), but the regulation of the cyclin D1 gene and its role in oestrogen-negative tumours has yet to be defined. Differential deregulation of several crucial cell cycle control molecules could possibly be implicated in hormone-independent breast cancer and may explain why a minor group of oestrogen receptor-negative patients in our study apparently had low cyclin E expression.

In summary, we have demonstrated large variations in cyclin E protein expression among tumours from patients with primary breast cancer, identified cyclin E as a new prognostic factor and could show a strong relationship between cyclin E protein abundance and oestrogen receptor-negative status, pointing to a possible role for cyclin E in the mechanisms responsible for oestrogen hormone-independent tumour growth. In our laboratory we currently study the pattern of expression of several important cell cycle regulators in oestrogen-positive and -negative tumours in the hope of getting a more coherent picture of the observed variations in cyclin expression and oestrogen receptor status, which in the future could possibly be applied to classify breast cancer according to the prevailing mechanisms of cell cycle deregulation.

**Acknowledgements**
We would like to thank Björn Tavelin for advice on statistical problems and for performing all statistical calculations, Anita Westman for collection and storage of breast tumour specimens, Göran Roos for critically reviewing the manuscript and Bodil Bäcklund for skilful technical assistance. This work was supported by grants from The Swedish Cancer Society 3448-B94-02XBB and The Lion's Cancer Research Foundation.

### References

ADNANE J, GAUDRAY P, SIMON M-P, SIMONY-LAFONTAINE J, JEANTEUR P AND THEILLET C. (1989). Proto-oncogene amplification and human breast tumor phenotype. *Oncogene*, **4**, 1389–1395.

ARNERLÖV C, EMDIN SO, ROOS G, ÅNGSTRÖM T, BJERSING L, ÄNGQUIST K-A AND JONSSON H. (1990). Static and flow cytometric DNA analysis compared to histologic prognostic factors in a cohort of stage T2 breast cancer. *Eur. J. Surg. Oncol.*, **16**, 200–208.

BUCKLEY MF, SWEENEY KJE, HAMILTON JA, SINI RL, MANNING DL, NICHOLSON RI, DeFAZIO A, WATTS CKW, MUSGROVE EA AND SUTHERLAND RL. (1993). Expression and amplification of cyclin genes in human breast cancer. *Oncogene*, **8**, 2127–2133.

BURTON K. (1968). Determination of DNA concentratiaons with diphenylamine. In *Methods in Enzymology*. Vol. 12, part B. Grossman L and Moldave K (eds) pp. 163–166. Academic Press: New York.

DOU Q-P, LEVIN AH, ZHAO S AND PARDEE AB. (1993). Cyclin E and cyclin A as candidates for the restriction point protein. *Cancer Res.*, **53**, 1493–1497.

DRAETTA GF. (1994). Mammalian G1 cyclins. *Curr. Opin. Cell Biol.*, **6**, 842–846.

DULIC V, LEES E AND REED SI. (1992). Association of human cyclin E with a periodic G1–S phase protein kinase. *Science*, **257**, 1958–1961.

DUTTA A, CHANDRA R, LEITER LM AND LESTER S. (1995). Cyclins as markers of tumor proliferation: immunocytochemical studies in breast cancer. *Proc. Natl Acad. Sci. USA*, **92**, 5386–5390.

FEICHTER GE, MUELLER A, KAUFMANN M, HAAG D, BORN IA, ABEL U, KLINGA K, KUBLI F AND GOERTTLER K. (1988). Correlation of DNA flow cytometric results and other prognostic factors in primary breast cancer. *Int. J. Cancer*, **41**, 823–828.

HARTWELL LH AND KASTAN MB. (1994). Cell cycle control and cancer. *Science*, **266**, 1821–1828.

HE J, ALLEN JR, COLLINS VP, ALLALUNIS-TURNER MJ, GODBOUT R, DAY RS AND JAMES CD. (1994). CDK4 amplification is an alternative mechanism to *p16* gene homozygous deletion in glioma cell lines. *Cancer Res.*, **54**, 5804–5807.

HERMAN ME AND KATZENELLENBOGEN BS. (1994). Alterations in transforming growth factor-$\alpha$ and -$\beta$ production and cell responsiveness during the progression of MCF-7 human breast cancer cells to estrogen-autonomous growth. *Cancer Res.*, **54**, 5867–5874.

HORWITZ KB. (1994). Plenary lecture: How do breast cancers become hormone resistant? *J. Steroid Biochem. Mol. Biol.*, **49**, 295–302.

HUNTER T AND PINES J. (1994). Cyclins and cancer II: cyclin D and CDK inhibitors come of age. *Cell*, **79**, 573–582.

KEYOMARSI K AND PARDEE A. (1993). Redundant cyclin overexpression and gene amplification in breast cancer cells. *Proc. Natl Acad. Sci. USA*, **90**, 1112–1116.

KEYOMARSI K, O'LEARY N, MOLNAR G, LEES E, FINGERT HJ AND PARDEE A. (1994). Cyclin E, a potential prognostic marker for breast cancer. *Cancer Res.*, **54**, 380–385.

KEYOMARSI K, CONTE JR. D, TOYOFUKU W AND FOX P. (1995). Deregulation of cyclin E in breast cancer. *Oncogene*, **11**, 941–950.

KOFF A, CROSS F, FISHER A, SCHUMACHER J, LEGUELLEC K, PHILIPPE M AND ROBERTS JM. (1991). Human cyclin E, a new cyclin that interacts with two members of the *CDC2* gene family. *Cell*, **66**, 1217–1228.

KOFF A, GIORDANO A, DESAI D, YAMASHAITA K, HARPER JW, ELLEDGE S, NISHIMOTO T, MORGAN DO, FRANZA BR AND ROBERTS JM. (1992). Formation and activation of a cyclin E-cdk2 complex during the G1 phase of the human cell cycle. *Science*, **257**, 1689–1694.

KORNBLAU SM, XU H-J, ZHANG W, HU S-X, BERAN M, SMITH TL, HESTER J, ESTEY E, BENEDICT WF AND DEISSEROTH AB. (1994). Levels of retinoblastoma protein expression in newly diagnosed acute myelogenous leukemia. *Blood*, **84**, 256–261.

LAMMIE GA, FANTL V, SMITH R, SCHUURING E, BROOKES S, MICHALIDES R, DICKSON C, ARNOLD A AND PETERS G. (1991). D11S287, a putative oncogene on chromosome 11q13, is amplified and expressed in squamous and mammary carcinomas and linked to BCL-1. *Oncogene*, **6**, 439–444.

LEACH FS, ELLEDGE SJ, SHERR CJ, WILLSON JKV, MARKOWITZ S, KINZLER KW AND VOLGELSTEIN B. (1993). Amplification of cyclin genes in colorectal carcinomas. *Cancer Res.*, **53**, 1986–1989.

MATSUSHIME H, ROUSSEL MF, ASHMUN RA AND SHERR CJ. (1991). Colony-stimulating factor 1 regulates novel cyclins during the G1 phase of the cell cycle. *Cell*, **65**, 701–713.

MEYER JS AND PROVINCE MA. (1994). S-phase fraction and nuclear size in long term prognosis of patients with breast cancer. *Cancer*, **74**, 2287–2299.

MOTOKURA T AND ARNOLD A. (1993). Cyclin D and oncogenesis. *Curr. Opin. Genet. Dev.*, **3**, 5–10.

MOTOKURA T, BLOOM T, KIM HG, JÜPPNER H, RUDERMAN, JV, KRONENBERG HM AND ARNOLD A. (1991). A novel cyclin encoded by a *bcl1*-linked candidate oncogene. *Nature*, **350**, 512–515.

MUSGROVE EA, HAMILTON JA, LEE CSL, SWEENEY KJE, WATTS CKW AND SUTHERLAND RL. (1993). Growth factor, steroid, and steroid antagonist regulation of cyclin gene expression associated with changes in T-47D human breast cancer cell cycle progression. *Mol. Cell. Biol.*, **13**, 3577–3587.

MUSGROVE EA, LEE CSL, BUCKLEY MF AND SUTHERLAND RL. (1994). Cyclin D1 induction in breast cancer cells shortens G1 and is sufficient for cells arrested in G1 to complete the cell cycle. *Proc. Natl Acad. Sci. USA*, **91**, 8022–8026.

OKAMOTO A, DEMETRICK DJ, SPILLARE EA, HAGIWARA K, HUSSAIN SP, BENNETT WP, FORRESTER K, GERWIN B, SERRANO M, BEACH DH AND HARRIS CC. (1994). Mutations and altered expression of $p16^{INK4}$ in human cancer. *Proc. Natl Acad. Sci. USA*, **91**, 11045–11049.

OTSUKI T, CLARK HM, WELLMAN A, JAFFE ES AND RAFFELD M. (1995). Involvement of *CDKN2*($p16^{INK4A}$/*MTS1* and $p15^{INK4B}$/*MTS2* in human leukemias and lymphomas. *Cancer Res.*, **55**, 1436–1440.

RESNITZKY D, GOSSEN M, BUJARD H AND REED SI. (1994). Acceleration of the $G_1$/S phase transition by expression of cyclin D1 and E with an inducible system. *Mol. Cell. Biol.*, **14**, 1669–1679.

SCHUURING E, VERHOEVEN E, TINTEREN H, PETERSE JL, NUNNINK B, THUNNISSEN FBJM, DEVILEE P, CORNELISSE CJ, VIJER MJ, MOOI WJ AND MICHALIDES RJAM. (1992). Amplification of genes within the chromosome 11q13 region is indicative of poor prognosis in patients with operable breast cancer. *Cancer Res.*, **52**, 5229–5234.

SIGURDSSON H, BALDETORP B, BORG Å, DALBERG M, FERNÖ M, KILLANDER D AND OLSSON H. (1990). Indicators of prognosis in node-negative breast cancer. *N. Engl. J. Med.*, **322**, 1045–1053.

SLAMON DJ, GODOLPHIN W, JONES LA, HOLT JA, WONG SG, KEITH DE, LEVIN WJ, STUART SG, UDOVE J, ULLRICH A AND PRESS MF. (1989). Studies of the HER-2/neu proto-oncogene in human breast and ovarian cancer. *Science*, **244**, 707–712.

SUTHERLAND RL, WATTS CKW AND MUSGROVE EA. (1993). Cyclin gene expression and growth control in normal and neoplastic human breast epithelium. *J. Steroid Biochem. Mol. Biol.*, **47**, 99–106.

TANDON AK, CLARK GM, CHAMNESS GC, CHIRGWIN JM AND McGUIRE WL. (1990). Cathepsin D and prognosis in breast cancer. *N. Engl. J. Med.*, **322**, 297–302.

TASSAN J-P, SCHULTZ SJ, BARTEK J AND NIGG EA. (1994). Cell cycle analysis of the activity, subcellular localization and subunit composition of human CAK (CDK-activating kinase). *J. Cell Biol.*, **127**, 467–478.

THEILLET C, ADNANE J, SZEPETOWSKI P, SIMON M-P, JEANTEUR P, BIRNBAUM D AND GAUDRAY P. (1990). *BCL-1* participates in 11q13 amplification forund in breast cancer. *Oncogene*, **5**, 147–149.

VAN AGTHOVEN T, VAN AGTHOVEN TLA, PORTENGEN H, FOEKENS JA AND DORSSERS LCJ. (1992). Ectopic expression of epidermal growth factor receptors induces hormone independence in ZR-75-1 human breast cancer cells. *Cancer Res.*, **52**, 5082–5088.

WANG TC, CARDIFF RD, ZUKERBERG L, LEES E, ARNOLD A AND SCHMIDT EV. (1994). Mammary hyperplasia and carcinoma in MMTV-cyclin D1 transgenic mice. *Nature*, **369**, 669–671.

WATTS CKW, SWEENEY KJE, WARLTERS A, MUSGROVE EA AND SUTHERLAND RL. (1994). Antiestrogen regulation of cell cycle progression and cyclin D1 gene expression in MCF-7 human breast cancer cells. *Breast C. Res. Treat.*, **31**, 95–105.

WEINBERG RA. (1995). The retinoblastoma protein and cell cycle control. *Cell*, **81**, 323–330.

WILLIAMS ME, SWERDLOW SH AND MEEKER TC. (1993). Chromosome t(11;14)(q13;q32) breakpoints in centrocytic lymphoma are highly localized at the bcl-1 major translocation cluster. *Leukemia*, **7**, 1437–1440.

XIONG Y, HANNON GJ, ZHANG H, CASSO D, KOBAYASHI R AND BEACH D. (1993). p21 is a universal inhibitor of cyclin kinases. *Nature*, **366**, 701–704.